# From Fragile Lives to Forensic Truth: Multimodal Forensic Approaches to Pediatric Homicide and Suspect Death

**DOI:** 10.3390/diagnostics15111383

**Published:** 2025-05-30

**Authors:** Kallirroi Fragkou, Ioannis Ketsekioulafis, Athina Tousia, Maria Piagkou, Flora Bacopoulou, Panagiotis Ferentinos, Pierre-Antoine Peyron, Eric Baccino, Laurent Martrille, Stavroula Papadodima

**Affiliations:** 1 Department of Forensic Medicine and Toxicology, School of Medicine, National and Kapodistrian University of Athens, 115 27 Athens, Greece; roifragou@gmail.com (K.F.); i.ketsekioulafis@gmail.com (I.K.); athinatousia@gmail.com (A.T.); 2 Department of Anatomy, School of Medicine, National and Kapodistrian University of Athens, 115 27 Athens, Greece; mapian@med.uoa.gr; 3 Center for Adolescent Medicine and UNESCO Chair in Adolescence Health Care, First Department of Pediatrics, School of Medicine, National and Kapodistrian University of Athens, Aghia Sophia Children’s Hospital, 115 27 Athens, Greece; fbacopoulou@med.uoa.gr; 4 Affective Disorders and Suicide Unit, 2nd Department of Psychiatry, “Attikon” University General Hospital, School of Medicine, National and Kapodistrian University of Athens, 124 62 Athens, Greece; pferentinos@med.uoa.gr; 5 Department of Legal Medicine, CHU Reunion, University of Reunion, 97400 Saint-Denis, France; pierre-antoine.peyron@chu-reunion.fr; 6 EDPFM, Department of Legal Medicine CHU Montpellier, University of Montpellier, 34000 Montpellier, France; e-baccino@chu-montpellier.fr (E.B.); laurent.martrille@chu-montpellier.fr (L.M.)

**Keywords:** child homicide, child abuse, infanticide, postmortem imaging, histological examination, forensic toxicology, postmortem microbiology, molecular autopsy, forensic investigation, sudden unexplained death in infancy (SUDI)

## Abstract

**Background:** Forensic investigation of child homicides presents unique challenges due to the vulnerability of children and the complexity of distinguishing between natural, accidental, and intentional manner of death. A multidisciplinary approach integrating traditional forensic methods with emerging technologies is crucial to ensure accurate diagnosis and effective legal outcomes. **Methods:** This review examines current and emerging forensic techniques used in neonate, infant, and older child homicide investigations. It highlights advancements in postmortem imaging, histological examination, microbiological analysis, toxicology, and molecular autopsy. **Results:** Traditional forensic autopsy remains the cornerstone of child homicide investigations, providing critical insights into external and internal injuries. Histological examination enhances diagnostic accuracy by detecting microscopic evidence of trauma and infectious diseases. Postmortem imaging techniques are complementary for better identifying fractures, soft tissue injuries, and vascular abnormalities. Forensic toxicology plays a key role in detecting poisoning, while postmortem microbiology aids in identifying infectious causes of death. Furthermore, advancements in molecular autopsy and genetic testing have significantly enhanced the identification of hereditary conditions linked to sudden unexplained deaths in children, especially in cases involving multiple child fatalities within the same family, where forensic investigations are needed to accurately differentiate between natural causes and potential criminal involvement. **Conclusions**: A multidisciplinary approach incorporating traditional autopsy with postmortem imaging, histological examination, toxicology, postmortem microbiology, and molecular autopsy is essential for comprehensive forensic analysis, promoting both justice and prevention of fatal child abuse/homicide. Future research should focus on standardizing forensic protocols and exploring the potential of artificial intelligence (AI) in forensic investigations.

## 1. Introduction

Child homicides are among the most distressing cases for forensic experts. Children are particularly vulnerable due to their physical fragility and inability to defend themselves. The circumstances surrounding their deaths require thorough investigation [1].

A “child” is generally defined as a human being between birth and puberty. This period encompasses various stages of development, including infancy, early childhood, middle childhood, and adolescence. Legally, the term often refers to a minor, which is typically considered a person younger than 18 years [2]. A neonate is defined as an infant during the first 28 days of life, whereas an infant is considered a child under one year of age [3].

Globally, child abuse and homicide rates are alarming. According to the United Nations Office on Drugs and Crime (UNODC), an estimated 205,153 children aged 0 to 14 years were victims of homicide worldwide between 2008 and 2017, averaging approximately 20,515 child homicides per year, but this figure may underestimate the actual number since many deaths from abuse are misclassified [4] due to factors such as declining autopsy rates. In the U.S., 1990 child fatalities due to abuse and neglect were reported in 2022, translating to a rate of 2.73 per 100,000 children and despite protective measures like mandatory reporting and Child Death Review (CDR) teams, many cases of child maltreatment go undetected until a forensic investigation occurs [5].

One of the greatest challenges lies in distinguishing a concealed infant homicide from a pathological death of unknown cause. Sudden Unexpected Deaths in Infancy (SUDI) represent the most frequently examined category of infant deaths in autopsies, yet it remains poorly understood. In 1968, Evans pointed out that infanticide has always existed in all countries, whether for religious reasons because of malformations found in the child, or for economic or psychiatric reasons on the part of the perpetrators [6]. In modern practice, even after extensive investigations and multiple ancillary tests, the cause of death may be established based on probability rather than absolute certainty. Despite these efforts, most SUDI cases remain unresolved [7]. These cases may spark extensive discussions about the potential involvement of criminal activity, particularly in specific situations, such as multiple infant deaths occurring within the same environment [8]. Sundwall et al. underline that “most child homicides are familial in nature and associated with investigational complexities that can lead to misdirection” [1].

The present review provides a comprehensive overview of both traditional and emerging forensic techniques used in homicide investigations involving children. By examining various forensic methodologies, from classic autopsy techniques to advanced DNA sequencing, this review highlights the importance of integrating new technologies to enhance the accuracy and effectiveness of investigations of these tragic deaths.

## 2. Investigation of the Death Scene and Autopsy

During forensic investigations of child homicides, reviewing the victim’s medical records and assessing the family’s socio-economic background, including siblings and parents, can provide crucial contextual information, given that several family situations have been reported to account for a notable percentage of child homicides [4].

A meticulous death scene investigation is equally critical in determining the cause and manner of death, particularly in subtle cases such as asphyxial deaths or instances where injuries are attributed to a fall while the infant was in the caregiver’s arms. One of the primary objectives of this investigation is to thoroughly document the environment in which the child was found, including the position of the body and any postmortem changes. In cases involving infants, specific livor mortis patterns may offer valuable forensic clues. For example, if livor mortis is observed as blanched only on the cheeks and tip of the nose, it may indicate that pressure was applied to the face, suggesting that the infant’s face remained pressed into the bedding postmortem. It is crucial to note that livor mortis remains “blanchable” or unfixed only during the initial 6 to 8 h after death. Careful inspection of the lividity may also reveal a change in the child’s position after death (for instance when parents claim not to have moved the body) [9].

Consequently, photographic documentation of lividity before transporting the infant is essential for reconstructing sleep position in sudden, unexpected infant deaths [10]. Additionally, investigators should carefully document nearby objects and the overall condition of the surroundings. Examining furniture and flooring may be particularly important in cases where caregivers attribute injuries to a fall. Items such as pillows, blankets, or plastic bags may have obstructed the child’s airway, while cribs, playpens, and sleeping areas should be assessed for compliance with safety standards [11].

Furthermore, technological devices at the scene can provide critical evidence to reconstruct the timeline of events and the caregiver’s actions before emergency services were contacted. For example, Brown et al. (2018) [12] reported a series of 20 asphyxial child homicides, including a case where apnea monitors contradicted the parents’ claims that they had not been in use, thereby exposing inconsistencies in their statements.

Autopsy is the cornerstone of any forensic investigation of child homicides. The literature strongly supports the usefulness of autopsy in these cases, especially when a violent death is suspected. During the autopsy, the goal extends beyond determining the cause of death to meticulously documenting every possible injury—external, internal, and microscopic—since even minor signs of trauma can be crucial for understanding the sequence of fatal events. Typically, the autopsy begins with a complete external examination, followed by an internal examination of the body, focusing particularly on the brain, abdomen, and thoracic cavity, as these are common sites of fatal injuries. Forensic pathologists must maintain vigilance for signs of blunt force trauma, sharp force injuries, or indications of neglect, such as malnutrition or dehydration [13]. Dissection of the face and extremities may reveal hidden soft tissue injuries that may not be visible during a standard external examination [14,15,16]. Removal of the eyes for further histological examination to reveal retinal hemorrhages has been considered the gold standard [17]; although in recent years, other non-invasive methods, such as postmortem fundus photography, have also been suggested [18].

## 3. Postmortem Imaging (PMI) Techniques

The continuous evolution of postmortem imaging (PMI) techniques has significantly enhanced forensic investigations, providing valuable tools that complement traditional autopsy procedures. Typically performed before a standard autopsy, these imaging modalities improve the examination of neonates, infants, and older children in cases of suspected homicide or abuse.

While the growing array of postmortem imaging techniques offers impressive capabilities, forensic applicability must be evaluated not only in terms of diagnostic performance but also in relation to cost, time-efficiency, and accessibility. Traditional radiography remains the cornerstone in the initial skeletal assessment of suspected child abuse cases, particularly for detecting long bone fractures. In resource-limited forensic settings, low-dose full-body digital X-ray systems such as Lodox^®^ Statscan have proven useful as rapid and affordable screening tools, enabling whole-body skeletal surveys with minimal radiation exposure. While not as detailed as CT, Lodox provides a valuable overview that can guide autopsy planning and support the identification of fractures or foreign bodies, especially in pediatric trauma cases. Moreover, its speed and ease of use make it ideal for emergency triage and for documenting injuries in neonates and infants when advanced imaging modalities are unavailable [19,20].

Advanced imaging modalities provide, however, distinct advantages in forensic analysis, enhancing diagnostic accuracy and comprehensiveness. In a study of 150 unexpected child deaths (aged two years and younger) between 2008 and 2018, postmortem imaging (usually skeletal survey, computed tomography (CT) head and/or a whole-body CT scan) revealed additional forensic findings in 34.0% of infants and 59.1% of children aged 13 to 24 months. Notably, in 11 cases with negative physical examination results, postmortem imaging successfully identified abusive injuries, while in 3 cases, it detected fractures that had been missing during the autopsy [21].

Indeed, Postmortem Computed Tomography (PMCT) is indispensable in identifying subtle fractures that may not be visible during conventional examinations and proves especially effective in challenging anatomical areas such as the costovertebral junction, sternum, and cervical spine. Furthermore, PMCT can distinguish different healing stages of fractures, playing a crucial role in cases of suspected chronic abuse. In addition to skeletal injuries, PMCT can detect hemorrhages, including subdural and epidural hematomas, as well as pneumothorax resulting from blunt force trauma—an injury often difficult to identify via traditional internal examinations [22,23]. Three-dimensional reconstructions in postmortem CT scans, using new technologies such as Global Illumination Rendering, enable us to better appreciate the characteristics of cranial fractures [24]. PMCT enables permanent 3D records for education and training, allows 3D-printed models for hands-on examination, enhances pathology visualization through magnification, and improves understanding of complex abnormalities [25]. Lung densitometry using postmortem CT may also provide a quantitative approach to the diagnosis of asphyxia, as a higher percentage of very low pulmonary density areas has been associated with certain asphyxial deaths, although the diverse pathophysiological mechanisms among asphyxia subgroups may limit the specificity of this finding [26].

Postmortem Magnetic Resonance Imaging (PMMRI) is particularly advantageous for soft tissue visualization, and is particularly effective in detecting congenital anatomical abnormalities, including brain malformations, renal anomalies, congenital heart defects, and skeletal dysplasias. Furthermore, PMMRI is particularly effective in visualizing spinal subdural hemorrhages and ligamentous injuries, which are often difficult to detect during standard autopsies. However, certain normal postmortem changes can be mistakenly interpreted as pathological conditions, such as bowel dilation being misidentified as an obstruction; whereas, recognizing typical postmortem changes, including fluid redistribution (such as subcutaneous edema, pleural and pericardial effusions, and ascites), can be difficult for radiologists who lack experience with autopsy imaging [23]. Moreover, it presents a significant financial burden for many centers [27].

Postmortem CT Angiography (PMCTA) employs radiopaque contrast agents to assess vascular injuries and abnormalities, though its application in pediatric forensic cases remains rare, except for stab wound injuries. Its use is limited by the need for specialized training, costly equipment, and extensive time requirements, making it less accessible for routine forensic applications [28].

Micro-CT offers high-resolution imaging down to micrometers, making it a valuable tool in postmortem forensics. It has demonstrated excellent diagnostic accuracy, especially for fetal hearts [28]. Micro-CT has been successfully used in fetal autopsies after miscarriage, providing high-resolution, non-invasive imaging. This technique reveals details often obscured by maceration or autopsy-related damage in small specimens [22]. In cases of suspected child abuse, micro-CT is particularly useful as it provides high-resolution images of bone microstructure, facilitating the detection of microfractures and aiding in distinguishing iatrogenic from non-accidental injuries [29].

Postmortem imaging may also be used in distinguishing stillbirth from a live birth with micro-CT offering detailed skeletal imaging, PMMRI detecting brain and internal organ abnormalities, and PMCT aiding in gestational age estimation by assessing long bone measurements and ossification centers [30]. A small-scale study suggested that PMMRI lung aeration is highly accurate in differentiating live birth from intrauterine fetal death, performing comparably to lung flotation tests. However, further validation in a larger perinatal cohort is needed [31].

The field of postmortem imaging continues to evolve with the development of innovative technologies. Three-Dimensional Surface Scanning (3DSS) provides high-resolution 3D models of external injuries (e.g., bruises and abrasions), enabling accurate documentation for forensic and legal purposes. While effective, 3DSS is limited to surface analysis and lacks the capability to visualize internal injuries [27].

Artificial intelligence (AI) is expected to significantly transform PMI by allowing for quicker and more objective analysis of imaging data. Machine learning algorithms can detect patterns in trauma or pathology that may not be obvious to human observers, improving forensic investigations’ accuracy and efficiency [32]. Recent systematic reviews, such as the one conducted by Ketsekioulafis et al. have identified potential applications of AI in human identification, postmortem interval estimation, and cause of death determination [33]. Despite these advancements, the integration of AI into standard forensic practice is still in its nascent stages. Challenges such as the need for extensive validation, the development of standardized protocols, and the acquisition of large, high-quality datasets must be addressed to ensure the reliability and accuracy of AI-driven tools in forensic contexts [33,34]. It is also self-evident that special protocols are required for the application of these technologies in pediatric forensic cases, due to the peculiarities of the child’s organism. In conclusion, AI can be an important tool in the investigation of child deaths, but targeted studies with a large volume of cases are required to develop individual technologies that will be sensitive and specific enough to be used in daily practice.

## 4. Histological Examination (HE)

Given the often subtle histopathologic changes seen on microscopic examination, the importance of a high-quality histotechnology laboratory, particularly with processing sampled brain sections, is stressed. Routine sections at baseline examination include hematoxylin- and eosin (H&E)-stained sections of all non-central nervous system (CNS) tissues and luxol fast blue/H&E-stained brain and spinal cord sections. Additional special stains are summarized in Table 1. Routine sampling has been suggested to include brain, dura and spinal cord, heart, lungs and major airways, liver, kidneys, hematopoietic (thymus, spleen, and bone marrow), endocrine (pituitary, thyroid gland, pancreas, and adrenal glands), and gastrointestinal (gastroesophageal junction, stomach, small intestine, and colon) tract [15].

A key question in the forensic examination of neonatal deaths is determining whether a neonate was born alive. If the neonate was stillborn, the possibility of neonaticide is ruled out. Assessments of developmental age and viability are essential in this process. Histological examination (HE) of lung tissue is beneficial, as lung morphology changes significantly when exposed to air. Stillborn neonates have dense alveolar spaces due to lack of oxygen exchange, while those that breathe exhibit expanded alveoli with air pockets visible under a microscope. However, relying solely on evidence of ventilation can be problematic. A significant amount of amniotic fluid aspiration, especially if stained with meconium, suggests severe intrauterine respiratory distress syndrome [37]. The assessment of lung tissue also reveals developmental stages—glandular, canalicular, saccular, and alveolar—indicating the neonate’s viability. Examining other organs like the brain and kidneys can provide further insights [37,38].

Hematoxylin and Eosin (H&E) stains can identify dystrophic axons but demonstrate changes only 18–24 h post injury. Silver staining methods, like Bielschowsky, can detect changes within 15–18 h. Immunohistochemical markers such as Amyloid Precursor Protein (APP), neurofilaments, Neuron-Specific Enolase (NSE), and others allow for axonal injury identification within a few hours [39]. In forensic investigations, it is crucial to determine whether axonal injury is traumatic or ischemic, but no definitive method currently exists to reliably differentiate between these causes [36].

In cases of suspected asphyxiation of infants and young children, external marks may be minimal or even absent due to their inability to resist such situations. Petechiae—tiny red or purple spots—are not always evident. In these cases, histological examination may provide some insight, although the findings remain debatable in the literature. Evidence of intra-alveolar hemorrhage and interstitial emphysema may indicate disrupted breathing efforts [12]. The presence of hemosiderin in pulmonary macrophages, identified through iron staining, has been suggested as a marker of previous asphyxial events or trauma associated with chronic child abuse. However, hemosiderin can also appear in various other conditions, such as pneumonia, heart failure, coagulopathies, or cases with ambiguous causes of death. Therefore, its presence is not considered diagnostic [35,40,41]. Hemosiderin-laden macrophages production typically begins around 24 to 36 h after a hemorrhagic incident and can persist for several weeks. This feature is particularly useful in identifying previous bleeding episodes, which is crucial in cases of brain trauma where earlier hemorrhages may be present before the fatal event. However, it does not provide precise information about the timing or cause of the bleeding [40].

When investigating the possibility of neglect, it is important to consider several factors. In addition to morphometric measurements taken during an autopsy, which help illustrate growth patterns and organ weights, histological examinations can provide valuable information. For instance, prolonged malnutrition can lead to atrophy of the liver and heart, while dehydration results in distinct histological patterns within kidney tissue. Hepatic microvesicular steatosis is often observed in pediatric cases of starvation due to protein deficiency. Microscopic examinations may reveal characteristics such as a “starry sky” appearance in the cytoplasm of thymus, calcification of Hassall’s corpuscles and replacement of thymic tissue with fibrofatty material, and secondary hemosiderosis of the spleen and liver [42,43,44]. Furthermore, histological evidence of untreated or chronic infections can indicate neglect, particularly when a caregiver fails to provide necessary medical care (medical neglect) [43].

In cases of poisoning and Munchausen Syndrome by Proxy (MSBP), prolonged exposure to toxic substances leads to distinctive cellular changes in organs such as the liver, kidneys, and brain. For example, liver tissue may show signs of fatty degeneration, a common response to chronic toxin exposure, while kidney tissue may display nephrotoxic changes, suggesting sustained contact with harmful substances [45,46].

Histological examination is crucial for identifying underlying medical conditions that may contribute to sudden natural deaths. These conditions include myocarditis, viral infections, interstitial pneumonia, bronchiolitis, and metabolic disorders [47]. A histological diagnosis can be achieved based on established consensus diagnostic criteria [48]. By recognizing these findings, forensic pathologists can differentiate between natural deaths and suspicious cases, leading to a clearer understanding of the actual cause of death. It is important to note that the literature includes cases of infant deaths where pathological findings indicated myocarditis or respiratory infections, which could explain the cause of death. However, in some cases, after a mother’s confession, these deaths were revealed to be homicides by asphyxiation. This underscores the reality that even a sick child can fall victim to homicide [12]. In some cases, they may be at even greater risk, as their increased crying and care demands can exacerbate parental stress and frustration [49].

## 5. Toxicological Examination

Toxicology plays a crucial role in child homicide investigations, as demonstrated by several case studies. Blood and urine are routinely collected for toxicological analysis; however, other biological materials, such as hair, bile, gastric content and vitreous humor, may also be preserved for further examination [50,51].

Cases of child poisoning can involve a diverse array of substances, ranging from household items to prescription medications and illicit drugs [50,51,52,53]. A study conducted by Gaw et al. (2023) [54], on fatal poisonings among U.S. children aged five years and younger, spanning from 2005 to 2018, revealed that opioids were the primary cause, followed by over-the-counter medications—a trend particularly notable in the United States. Most poisoning incidents occurred at home, often when the child was under non-parental supervision, with infants (less than one year old) being particularly vulnerable. Deliberate poisonings were more common in children under two years old and frequently associated with neglect or maltreatment. Moreover, forensic investigations have detected elevated blood concentrations of antidepressants and antipsychotics, such as mirtazapine and alimemazine, suggesting their potential involvement in intentional poisoning cases [55,56].

Bonsignore et al. reported a 32-month-old child who died from methadone intoxication administered by his drug-dependent parents. Toxicological analysis revealed lethal methadone levels in the child’s blood and hair, indicating repeated exposure, which was also found in his younger sibling [57]. In another instance, a 15-day-old boy died from acute mirtazapine poisoning, with levels in his blood significantly higher than therapeutic doses, suggesting intentional administration by his mother, who was suffering from postpartum psychosis [55]. Barros et al. reported on a nurse who attempted to kill eleven newborns by administering large doses of morphine and other drugs. Her actions, captured on camera, revealed psychopathy and Munchausen syndrome by proxy [58]. In cases of Munchausen Syndrome by Proxy (MSBP), perpetrators may administer various substances, such as sedatives or emetics, to create symptoms that mimic a natural illness and necessitate medical attention. Vennemann et al. (2005) [59] reported cases where caregivers with MSBP poisoned their young victims using a range of substances. Among the drugs identified were theophylline, which was linked to toxic levels in a child presenting with agitation and convulsions, as well as carbamazepine, clonazepam, and phenobarbital, all of which caused severe neurological symptoms. Clozapine, used to sedate children, was found during postmortem examinations in some cases. Other substances included doxylamine and propyphenazone, which, in one instance, resulted in recurrent neurological and gastrointestinal symptoms [59]. Hair analysis is valuable in these cases, particularly in situations involving suspected long-term poisoning [56].

A unique case of snake envenomation has also been reported as a means of child homicide. A father used an Egyptian cobra to repeatedly bite his three daughters, aged four, six, and nine, causing their deaths [60]. The forensic investigation of such cases requires specialized techniques to detect venom proteins and toxins [61,62,63].

Metabolism and physiological response to toxins in children, particularly infants, differ significantly from adults. Children’s immature metabolic systems can alter the effects of toxic substances, often leading to prolonged presence and impact of substances in their bodies. Consequently, toxicologists must calibrate their methodologies to account for these developmental factors. Standard adult toxicological ranges do not apply, and this discrepancy complicates the interpretation of toxicological data in pediatric cases. Determining what constitutes a lethal dose is challenging, especially given the lack of pediatric-specific toxicology reference ranges in many cases. Additionally, small body size and rapid metabolic rates in infants necessitate careful sampling, as minute quantities of toxins can reach fatal levels more quickly. Moreover, because biological samples from infants are typically small in volume, forensic toxicologists must be skilled in maximizing data from limited sample quantities, which calls for high-precision testing methodologies. These limitations mean that toxicologists must balance accuracy and sensitivity when interpreting toxicological results to avoid misclassification of accidental versus intentional poisoning [64,65].

For years, methods such as gas chromatography/mass spectrometry (GC/MS) and liquid chromatography/mass spectrometry (LC-MS) have been the norm for identifying and evaluating substances. Enzyme-linked immunosorbent assay (ELISA) is widely used for initial screenings due to its rapid results and capability to detect specific drug classes. Positive results are typically confirmed using GC-MS or LC-MS. In recent years, liquid chromatography-tandem mass spectrometry (LC-MS/MS) and gas chromatography-tandem mass spectrometry (GC-MS/MS) have been rendered the most widely used techniques for the quantification of drugs and poisons in biological specimens [66,67,68].

With the ongoing technological advances, new techniques have emerged, like Surface-Enhanced Raman Spectroscopy (SERS) and Fourier Transform Ion Cyclotron Resonance Mass Spectrometry (FTMS), enhancing trace detection and molecular analysis. Capillary Electrophoresis (CE) offers rapid separation of diverse compounds. Continuous advancements aim to improve sensitivity, accuracy, and applicability in forensic investigations [69,70].

A particularly promising innovation in forensic toxicology is the application of metabolomics. This approach involves analyzing the complete set of small molecules within a biological sample, offering a comprehensive view of metabolic disturbances caused by toxicants. Metabolomics can identify biochemical changes induced by toxic substances in a child’s system, helping to detect exposure patterns over time and providing crucial insights into chronic poisoning or repeated low-dose exposure. However, further research is necessary to fully validate its potential before metabolomics can be reliably used as evidence in medicolegal investigations [71].

## 6. Microbiological Examination

Forensic microbiology, also referred to as postmortem microbiology (PMM) when applied to death investigations, has emerged as a promising and rapidly advancing field within forensic science. This discipline enhances traditional investigative methods by addressing critical gaps in knowledge, particularly in cases of unexplained or suspicious deaths. It focuses on the analysis of microorganisms as forensic tools to help determine the cause and time of death, especially in cases where infections may have contributed to the fatal outcome. The identification of pathogenic microbes is increasingly recognized as essential in medicolegal investigations, aiding in the clarification of infection-related deaths, the confirmation of clinical suspicions, the assessment of antimicrobial treatment efficacy, the detection of emerging pathogens, and the correction of diagnostic errors [72,73,74]. Beyond its role in investigating infection-related fatalities, forensic microbiology also has expanding applications in crime scene analysis, biocrimes, bioterrorism, trace evidence examination, and even geolocation or source tracking in forensic casework, contributing to a more comprehensive and multidisciplinary approach to death investigations [75,76].

Despite the growing recognition of PMM in forensic investigations, challenges remain, particularly the lack of standardized sampling methods. The Explained Sudden Death of Infancy (SDI) panel identifies infections (46%), accidental causes (15%), congenital anomalies (14%), non-accidental injury (13%), and other causes (12%), as leading contributors to death. Given the high likelihood of infection and potential for non-accidental injury, microbiological sampling is crucial during differential diagnosis, including bacteriological and virological testing [77].

Postmortem bacteriological testing is crucial for identifying infections that lack visible signs, such as those causing systemic inflammation or sepsis. Pathogens like Staphylococcus aureus and Escherichia coli can lead to septic shock without observable lesions, making traditional examinations inadequate. Botulism, caused by Clostridium botulinum, exemplifies this challenge; it often presents subtle symptoms like muscle weakness and respiratory distress, progressing to death without external signs [78].

One hypothesis suggests that sudden infant death syndrome (SIDS) may be linked to transient bacteremia, which lasts less than 20 min and could explain the lack of histological changes. This is particularly relevant for infants aged two to three months, who have immature immune systems and low anti-toxin antibodies. Although no single theory fully explains the causes of unexplained SDI, research indicates that infections may contribute in some cases. The “common bacterial toxin hypothesis” posits that toxins from the infant’s own microbial flora could trigger excessive immune responses or affect neural and muscle membranes, leading to sudden death. Additionally, viral infections may act as stressors under the “triple risk hypothesis” [78,79,80].

Viral infections like cytomegalovirus and herpes simplex may not show clear symptoms, yet they can significantly contribute to mortality. Among children, respiratory infections are the leading cause of virus-related sudden death, often resulting in multiorgan failure. Inconsistent virological testing and methodological biases have limited our understanding of viral roles in sudden deaths, emphasizing the need for thorough microbial analysis in these cases [73,78,81].

The timing of postmortem sample collection is crucial for accurate forensic analysis. Ideally, samples should be taken immediately after death to minimize contamination. However, if the body is refrigerated, valuable results can still be obtained within 24 h postmortem. Delays beyond this period or non-refrigerated conditions increase the risk of bacterial overgrowth, complicating findings [72].

In infant death investigations, pre-autopsy and during-autopsy sampling are critical for diagnostic accuracy. Pre-autopsy sampling involves collecting sterile samples of blood, urine, cerebrospinal fluid (CSF), and swabs from the nasopharynx, trachea, and rectum. These samples are essential for detecting infections such as sepsis, meningitis, or gastrointestinal infections, with the goal of ensuring that findings reflect antemortem conditions rather than contamination from the autopsy procedure. During the autopsy, samples are collected from internal organs and tissues to confirm infection sites and understand pathogen dissemination. Common tissues sampled include the lungs, liver, spleen, brain, meninges, and bone marrow [74,77,78,82,83]. The types of samples collected in infant death investigations are described in Table 2.

Diagnostic methods for identifying pathogens include traditional microbial cultures, Gram staining, and advanced molecular techniques such as polymerase chain reaction (PCR) and next-generation sequencing (NGS). PCR, for example, is especially valuable for detecting respiratory and cardiotropic viruses in frozen autopsy tissues. These diagnostic methods have successfully identified various pathogens responsible for sudden death, including adenovirus, influenza viruses, respiratory syncytial virus (RSV), and cytomegalovirus (CMV). Additionally, advanced tools like 16S rRNA sequencing, NGS, and targeted nucleic acid amplification tests (NAATs) have enhanced the detection of complex microbial populations and rare pathogens in postmortem samples, thereby complementing traditional methods [72,77,78,82,83,84].

The thanatomicrobiome—the collection of microorganisms that thrive in the body after death—can offer insights into the postmortem interval (PMI), or the time since death. These microorganisms are vital to the decomposition process, encouraging forensic researchers to study their composition for applications in forensic science. By analyzing microbial changes in specific tissues, scientists may improve the accuracy of death investigations and establish a timeline of death based on microbial evidence [83,85].

Challenges in PMM persist despite advancements. Bacterial growth in postmortem samples can arise from true infections, postmortem translocation, agonal spread, or contamination. While contamination cannot be fully eliminated, it can be reduced through strict protocols. Agonal spread remains a theoretical concept, and postmortem translocation and contamination are influenced by diet, lifestyle, and geography. Standardized procedures and refrigeration are crucial to minimizing these issues. As forensic microbiology evolves, it will increasingly enhance pediatric forensic science, aiding in cause-of-death determinations and improving public health outcomes [73,74,75,78,80,83].

## 7. Complementary Examinations

Postmortem biochemistry (PMB) involves analyzing blood, cerebrospinal fluid, and vitreous humor to detect abnormalities such as electrolyte imbalances, toxic substances, and metabolic dysfunctions. PMB may aid in identifying metabolic disorders (fatty acid oxidation defects, organic acidemias, and urea cycle defects can be revealed through blood and CSF analysis), infections (elevated markers such as C-reactive protein), electrolyte imbalances, and endocrine disorders such as hyperglycemia or adrenal insufficiency, indicated by glucose and cortisol levels. PMB should ideally be performed within the first 2 h after death to minimize changes due to decomposition or autolysis. Certain fluids like vitreous humor remain relatively stable longer, making them valuable for analysis in delayed cases [84,86].

Recent advances in molecular biology have made molecular autopsy a valuable tool, especially when traditional autopsies cannot determine the cause of death. This method integrates genetic testing and forensic pathology to investigate hereditary causes, focusing on cardiac and metabolic anomalies that can lead to sudden unexplained deaths in infants and children. Many pediatric deaths from sudden cardiac events are linked to specific genetic mutations related to cardiac arrhythmias and cardiomyopathies [87,88].

There are two primary testing approaches: Targeted Genetic Panels, which focus on specific genes associated with these disorders, and Whole Exome Sequencing (WES), which analyzes all protein-coding regions of genes. While WES offers a comprehensive view and has a higher chance of identifying pathogenic variants, it may also uncover variants of uncertain significance (VOUS). This can complicate case conclusions in forensic settings [89].

The technical, scientific, ethical, and legal dilemmas associated with genetic testing are beyond the scope of this review. However, it is essential to emphasize that accurate interpretation of genetic testing results can prevent forensic pathologists from misinterpreting a sudden infant or child death as suspicious, particularly in cases involving multiple sudden deaths within the same family.

## 8. Conclusions

The forensic investigation of homicides involving neonates, infants, and children necessitates a multidisciplinary approach that fuses traditional techniques with cutting-edge technologies, ensuring a comprehensive analysis of each case. Autopsies serve as the cornerstone of forensic examinations, yielding essential insights through thorough documentation of both external and internal injuries. Advanced imaging techniques, including PMCT and PMMRI are useful for detecting subtle fractures, hidden injuries, and vascular abnormalities often missed during traditional autopsy. Histological examination further augments diagnostic accuracy by revealing subtle microscopic changes and identifying various forms of trauma, metabolic disorders, or infectious conditions. Toxicological analyses are indispensable for detecting poisoning, substance misuse, and chronic exposure, often uncovering causes of death that might otherwise remain undetected. Postmortem microbiology is crucial for identifying infections or underlying pathologies, especially when histological evidence is not available. Complementary examinations such as postmortem biochemistry and molecular autopsies yield critical information about metabolic disorders, genetic predispositions, and biochemical imbalances. Together, these methods form a robust framework for tackling the complexities of child homicide cases, providing clarity amid diagnostic challenges and promoting the pursuit of justice and the prevention of such tragic events.

## Figures and Tables

**Table 1:** Stain types for histopathologic purposes in cases of infant and child deaths [35,36].

Stain Type	Application Area	Purpose/Goal
Hematoxylin and Eosin (H&E)	All tissues	Routine examination of tissue samples (hypoxic-ischemic lesions, edema, hemorrhages, etc.)
Luxol Fast Blue/H&E	Brain and spinal cord	Detection of lesions in the central nervous system (CNS)—evaluates myelination in white matter and identifies demyelination, a common finding in chronic hypoxia
Iron Stain	Lung	Detection of hemosiderin and hemorrhages
Periodic Acid-Schiff (PAS)	Myocardium, kidney, and liver	Detection of glycogen, mucins, and basement membrane abnormalities.
Elastic Trichrome	Myocardium, kidney, and liver	Evaluation of connective tissue and elastic fibers.
Grocott Methenamine Silver (GMS)	Tissues with suspected infection	Identification of fungal infections.
PAS with Diastase (PASD)	Tissues with suspected infection	Differentiation of glycogen from other periodic acid-reactive substances.
Gram Stain	Tissues with suspected infection	Identification of bacterial infections.
Ziehl-Neelson (ZN) Stain	Tissues with suspected infection	Detection of acid-fast bacilli (e.g., tuberculosis).
Glial Fibrillary Acidic Protein (GFAP)	Brain and spinal cord	Detection of gliosis in neuropathological examination/indicator of metabolic, toxic, or degenerative disorders
NeuN	Brain	Identification of focal cortical dysplasia.
CD68	Brain and spinal cord	Detection of microglial activation (e.g., microglial nodules, particularly useful in evaluating inflammation.).
Beta-Amyloid Precursor Protein (βAPP)	Brain	Detection of axonal swellings caused by oxygen deprivation or trauma (ischemic or traumatic axonal injury)
Elastic, Trichrome, and Iron Stains	Brain (intracranial hemorrhage)	Accurate dating of intracranial hemorrhages.
Tyrosine Hydroxylase and GABA Receptor Staining	Brain (locus coeruleus/brain stem)	Evaluation of neurotransmitter abnormalities, such as serotonin deficits or dysfunction in GABAergic systems associated with immature respiratory and autonomic function in SIDS

**Table 2:** Samples and techniques used in cases of infant deaths.

Sample Type	Techniques Used	Potential Findings
Blood	Microbial culture Gram staining PCR 16S rRNA sequencing	*Escherichia coli* (sepsis) - *Streptococcus pneumoniae* (septicemia)
Cerebrospinal Fluid (CSF)	Microbial culture Gram staining PCR 16S rRNA sequencing	*Streptococcus pneumoniae*, *Neisseria meningitidis* (bacterial meningitis)
Pulmonary Fluid	Microbial culture PCR	*Staphylococcus aureus*, *Klebsiella pneumonia* (pneumonia)
Pleural Fluid	Microbial culture Gram staining PCR	*Pseudomonas aeruginosa*, *Streptococcus pneumoniae*
Peritoneal Fluid	Microbial culture PCR	*Escherichia coli*, *Bacteroides fragilis* (abdominal infections)
Urine	Microbial culture PCR	*Escherichia coli*, *Klebsiella* spp. (urinary tract infections)
Feces	Microbial culture PCR	*Clostridium difficile*, *Salmonella* spp. (gastrointestinal infections)
Tissue Biopsies	Microbial culture PCR	Evidence of localized infections or inflammation (*Staphylococcus aureus*, viral myocarditis)

## Abbreviations

The following abbreviations are used in this manuscript:

AI	Artificial Intelligence
APP	Amyloid Precursor Protein
CE	Capillary Electrophoresis
CDR	Child Death Review
CNS	Central Nervous System
CSF	Cerebrospinal Fluid
ELISA	Enzyme-Linked Immunosorbent Assay
FTMS	Fourier Transform Ion Cyclotron Resonance Mass Spectrometry
GC/MS	Gas Chromatography/Mass Spectrometry
GFAP	Glial Fibrillary Acidic Protein
GMS	Grocott Methenamine Silver
HE	Histological Examination
H&E	Hematoxylin and Eosin
LC-MS	Liquid Chromatography/Mass Spectrometry
LC-MS/MS	Liquid Chromatography–Tandem Mass Spectrometry
MSBP	Munchausen Syndrome by Proxy
NAAT	Nucleic Acid Amplification Test
NGS	Next-Generation Sequencing
NSE	Neuron-Specific Enolase
PAS	Periodic Acid–Schiff
PASD	Periodic Acid–Schiff with Diastase
PCR	Polymerase Chain Reaction
PM	Postmortem
PMB	Postmortem Biochemistry
PMCT	Postmortem Computed Tomography
PMCTA	Postmortem CT Angiography
PMI	Postmortem Imaging
PMMRI	Postmortem Magnetic Resonance Imaging
PMM	Postmortem Microbiology
SIDS	Sudden Infant Death Syndrome
SUDI	Sudden Unexplained Death in Infancy
SERS	Surface-Enhanced Raman Spectroscopy
SDI	Sudden Death of Infancy
VOUS	Variants of Uncertain Significance
WES	Whole Exome Sequencing
ZN	Ziehl–Neelsen
